# Supplementary material for: Genre-aware user profiling using duration count matrices: A novel approach to enhancing content recommendation systems
Source: PLoS One. 2025 Apr 1;20(4):e0312520. doi: 10.1371/journal.pone.0312520 (PMC11960937; doi:10.1371/journal.pone.0312520)
Supplement: Attachment — Submitted filename: Response to Review Letter (6).docx [file pone.0312520.s001.docx]

**Original Manuscript ID:** EMID:af33cb82f41d736b

**Original Article Title:** “Genre-Aware User Profiling using Duration Count Matrices: A Novel Approach to Enhancing Content Recommendation Systems”

To: JISA Edutor

Re: Response to reviewers

Thank you for allowing a resubmission of our manuscript with an opportunity to address the reviewers’ comments.

We are uploading:

(a)	A point-by-point response to the reviewer 1 comments (below).

(b)	Updated manuscript with red text highlighting the changes.

(c)	Updated manuscript without highlights (PDF main document).

The authors would like to thank all the reviewers for their constructive feedback. Please let us know if you require any further changes from our side.

Best Regards

--------------------

Best Regards
Asadullah Shaikh, Ph.d., (University of Southern Denmark)
(www.asadshaikh.com)
Professor,
Head of Research &
Coordinator Seminars and Training,
College of Computer Science,
Najran University, Saudi Arabia
Adr. Saudi Arabia - Najran - King Abdulaziz Road

**Reviewer 1**

**Dear Reviewer,**

We are grateful for your time and valuable suggestions. We understand that these suggestions have really uplifted our manuscript. Considering your suggestions, we improved our manuscript. Following are the responses to each suggestion.

______________________________________________________________________________

**Comment 1:** The gap of the previous works in the research should be examined and then the proposed idea should be presented based on it.

**Author Response**: Addressed. Please see the section 1 page 1 and section 2 page 2 with yellow highlights.

**Actions:**

______________________________________________________________________________

**Comment 2:** Data labeling leads to a decrease in the accuracy of the recommender system. Based on the labeled data that exists in the data set, the classification should be performed.

**Author Response**: In our study, we proposed two classification techniques, DCM-UP (Duration-Count Matrix-based User Profiling) and DCM-US (Duration-Count Matrix-based User Similarity). When evaluating DCM-US, both default and tuned hyper parameters (HPs) yielded impressive results, with accuracies ranging from 97% to 98%. However, with DCM-UP, default HPs outperformed tuned ones, achieving an accuracy of 89%. This discrepancy aligns with findings from previous research suggesting that many datasets perform optimally with default HP values, particularly when using decision trees (DT), depending on dataset characteristics. Our approach involved employing both grid search and iterative methods to identify the best HP configurations. Despite these efforts, there are several reasons why tuned HPs may not perform well. These are:

Fine-tuning hyperparameters can lead to overfitting, where the model specializes too much on the training data, losing its ability to generalize. This can happen because fine-tuning may push the model to capture noise rather than true patterns. Additionally, it can introduce bias, favouring certain features over others, reducing generalization performance. Moreover, it may cause instability, where small adjustments lead to significant prediction variations. This instability makes the model less reliable. Additionally, it may also increase computational costs and training time, especially with exhaustive search methods.

**Actions:**

**Comment 3:** What are the reasons for choosing classifiers of decision tree and random forest among the existing classifiers? According to the problem, classifications based on probabilities can be used (such as Naive Bayes).

**Author Response:** The preference for decision tree classifiers over other machine learning algorithms like Naive Bayes, KNN, linear regression and SVM in user behavior prediction arises from their interpretability and versatility. In addition to applying all these models to our dataset, it's essential to consider their strengths and limitations.

Decision trees offer clear decision paths and perform well with both numerical and categorical data, achieving high accuracy rates, such as 90–98%, in our techniques. On the other hand, Naive Bayes and KNN might struggle with more complex data, leading to lower accuracy, often below 50%. Meanwhile, SVM, although powerful, can be really slow and hard to use with big sets of data, as shown by the long time it took to finish. In contrast, linear regression struggles with nonlinear user behavior data relationships due to its linear assumptions.

**Actions:**

**Comment 4:** The novelty of the paper is limited. Many proposed techniques have been studied/applied before in previous research.

**Author Response**: In the domain of video recommendation techniques, it's acknowledged that various methods exist to support personalized recommendations. However, the majority of these techniques relies on explicit features or feedback, such as reviews, star ratings, comments, and likes, to predict user behavior and recommended videos accordingly. Additionally, some techniques leverage implicit behaviors like retweets, shares, view-time, hash tags, visual contents, and demographic information to predict both short-term and long-term user behaviors for video recommendations, as illustrated in Table 11.

In contrast, the novelty of our proposed technique lies in its distinctive approach. We utilize an implicit feature known as "Watch-time duration" to predict user's long-term behaviors effectively. For recommendation purposes, we construct a matrix based on video genres and their corresponding watch-time durations. This matrix forms the foundation for recommending videos that closely align with users' preferences and behaviors.

Furthermore, our proposed technique benefits from its reliance on a fundamental aspect of user engagement: watch-time duration. Research has shown that watch-time duration is a robust indicator of user interest and satisfaction with video content. By prioritizing this metric in our recommendation algorithm, we ensure that recommended videos are not only relevant but also likely to capture and retain users' attention.

Moreover, our proposed approach capitalizes on the growing trend of personalized content recommendations in the digital streaming-platform. As streaming platforms continue to expand and compete for users' attention, the ability to deliver videos for recommendations based on complex user preferences becomes increasingly crucial. By incorporating watch-time duration into our recommendation model, we address this need for personalized content discovery, enhancing users' overall viewing experience.

Ultimately, the integration of watch-time duration into our recommendation algorithm represents a forward-thinking approach to video recommendation systems. By focusing on a reliable and actionable metric of user engagement, we not only improve the accuracy of our recommendations but also encourage greater user satisfaction and retention on video-streaming platforms.

**Actions:**

**Comment 5:** Clustering algorithms that determine the number of clusters are a suitable option compared to k-means algorithm. How is the number of clusters determined in the proposed method?

**Author Response**: The choice of K-means clustering in the DCM technique is justified by its compatibility with numeric features like watch-time durations, ensuring effective clustering. Its computational efficiency and scalability make it ideal for large datasets, providing easily interpretable clusters. Moreover, the structural alignment between the DCM's user-genre matrix and K-means' data organization enhances understanding and prediction accuracy. Furthermore, K-means' scalability addresses the challenge of expanding behavior datasets over time, accommodating large volumes of data without significant computational burden.

Addressed. Please see the section 4.4 page 12 and 13 with yellow highlights.

**Actions:**

**Comment 6:** How is the distance criterion set in the clustering algorithm?

**Author Response**: In the context of my research utilizing the K-means clustering algorithm, the Euclidean distance metric was deemed most appropriate for several reasons. Firstly, the Euclidean distance is widely accepted and employed in clustering algorithms due to its simplicity and effectiveness. Secondly, it aligns well with the nature of my dataset, which consists of numeric features such as watch-time durations. This metric provides a clear measure of dissimilarity between user behavior patterns, crucial for effective clustering.

Addressed. Please see the section 4.4 page 12 with yellow highlights.

**Actions:**

**Comment 7:** Comparison with previous valid works should be done in the evaluation section of the proposed method. Error-based measures should also be evaluated in this section.

**Author Response**: Addressed. Please see the section 6.4 pages 26, 27 & 32 with yellow highlights. For comparison table, please see the section 6.4 pages 28, 29 & 30.
